# A Good Practice–Compliant Clinical Trial Imaging Management System for Multicenter Clinical Trials: Development and Validation Study

**DOI:** 10.2196/14310

**Published:** 2019-08-30

**Authors:** Youngbin Shin, Kyung Won Kim, Amy Junghyun Lee, Yu Sub Sung, Suah Ahn, Ja Hwan Koo, Chang Gyu Choi, Yousun Ko, Ho Sung Kim, Seong Ho Park

**Affiliations:** 1 Department of Radiology Asan Medical Center University of Ulsan College of Medicine Seoul Republic of Korea; 2 Research Institute of Radiology Asan Medical Center University of Ulsan College of Medicine Seoul Republic of Korea; 3 Asan Image Metrics Clinical Trial Center Asan Medical Center, University of Ulsan College of Medicine Seoul Republic of Korea; 4 Procuratio, Co Ltd Seoul Republic of Korea

**Keywords:** clinical trial, information technology, diagnostic imaging, regulation, computerized system validation

## Abstract

**Background:**

With the rapid increase in utilization of imaging endpoints in multicenter clinical trials, the amount of data and workflow complexity have also increased. A Clinical Trial Imaging Management System (CTIMS) is required to comprehensively support imaging processes in clinical trials. The US Food and Drug Administration (FDA) issued a guidance protocol in 2018 for appropriate use of medical imaging in accordance with many regulations including the Good Clinical Practice (GCP) guidelines. Existing research on CTIMS, however, has mainly focused on functions and structures of systems rather than regulation and compliance.

**Objective:**

We aimed to develop a comprehensive CTIMS to meet the current regulatory guidelines and various required functions. We also aimed to perform computerized system validation focusing on the regulatory compliance of our CTIMS.

**Methods:**

Key regulatory requirements of CTIMS were extracted thorough review of many related regulations and guidelines including International Conference on Harmonization-GCP E6, FDA 21 Code of Federal Regulations parts 11 and 820, Good Automated Manufacturing Practice, and Clinical Data Interchange Standards Consortium. The system architecture was designed in accordance with these regulations by a multidisciplinary team including radiologists, engineers, clinical trial specialists, and regulatory medicine professionals. Computerized system validation of the developed CTIMS was performed internally and externally.

**Results:**

Our CTIMS (AiCRO) was developed based on a two-layer design composed of the server system and the client system, which is efficient at meeting the regulatory and functional requirements. The server system manages system security, data archive, backup, and audit trail. The client system provides various functions including deidentification, image transfer, image viewer, image quality control, and electronic record. Computerized system validation was performed internally using a V-model and externally by a global quality assurance company to demonstrate that AiCRO meets all regulatory and functional requirements.

**Conclusions:**

We developed a Good Practice–compliant CTIMS—AiCRO system—to manage large amounts of image data and complexity of imaging management processes in clinical trials. Our CTIMS adopts and adheres to all regulatory and functional requirements and has been thoroughly validated.

## Introduction

### Background

In the last decade, the number of clinical trials including multicenter trials has increased worldwide and consequently, the related clinical data have increased and grown more complex in many types of sources. Furthermore, medical imaging involvement in the recent clinical trials is another main reason for increasing the intricacies of clinical data [1-3]. These increased clinical data help develop information technology (IT) systems such as electronic data capture (EDC) systems or clinical trials management systems in order to access and collect data efficiently [4]. As a result, a separate dedicated IT system for imaging data is required to manage data and control any risk from the clinical trials because of increased medical imaging usage for various imaging endpoints in multicenter trials.

Since image biomarkers have been used in a variety of clinical trials [5], the US Food and Drug Administration (FDA) has continuously emphasized on the importance of tumor response on medical images for regular approval of oncology drugs [6,7]. To provide instructions for the standardization process of image biomarker use in clinical trials, the FDA issued “Clinical trials Imaging Endpoint Process Standards Guidance for Industry” in 2018 (hereafter referred to as 2018 FDA imaging guidance) [8].

According to the FDA imaging guidance, the standardization of clinical trials imaging endpoints describes processes to manage imaging acquisition, quality check, anonymization, transfer, archive, quantitative image analysis, and independent blinded image review by multiple readers [9]. These processes must also comply with regulations and guidelines of clinical trials, including the Good Clinical Practice (GCP) guidelines [10] and Health Insurance Portability and Accountability Act (HIPAA) [8]. Both imaging processes and data should follow standard global data formats outlined by the Clinical Data Interchange Standards Consortium (CDISC), the Health Level 7, and the Digital Imaging and Communications in Medicine (DICOM) [9]. Furthermore, a diversity of parties such as pharmaceutical companies, contract research organizations (CROs), sites or hospitals, and central imaging readers should access the updated data in real time, especially for multicenter trials, to track the study and review the imaging data. These factors have necessitated image data integration and management, resulting in the development of an IT system for clinical trial imaging [11].

Recently, several different IT platforms have been developed specifically for clinical trials that enable integrated imaging data management and efficient imaging workflows [1,9]. Such platforms are referred to as Clinical Trial Imaging Management Systems (CTIMS). A typical CTIMS contains a Web-based EDC system with either a standalone or Web-based DICOM image viewer. CTIMS are expected to maintain clinical trial regulatory compliance, use globally standardized data formats, and be validated with GxP-based protocols, where G stands for “Good”; P stands for “Practice”; and x stands for the regulatory fields such as good clinical, laboratory, or manufacturing practices [2,12].

The GxP is a collection of quality guidelines and regulations to ensure medical product safety; their intended use; and quality processes during clinical development, manufacturing, and distribution [12-14]. Although many guidelines and regulations for clinical trials are issued, the most important guideline for the CTIMS and EDC system is GCP, which is provided by the International Conference on Harmonization (ICH) [10]. However, the ICH-GCP does not cover the detailed requirements for computerized systems. Instead, regulatory agencies [15,16] and international societies [17,18] provide standards to build and use computerized systems in clinical trials. Hence, the term “GxP compliance” includes various guidelines, including GCP, Good Clinical Laboratory Practice, and Good Manufacturing Practice.

### Objectives

Many studies have focused on CTIMS [9,11,19], but no study has thus far comprehensively discussed the regulations and guidelines related to CTIMS, particularly with regard to GxP compliance. Therefore, we developed a CTIMS, named the AiCRO system, designed to comprehensively meet the current regulatory guidelines and perform GxP-based computerized system validation. In this article, we aim to describe the methods, considerations, and recommendations for the development and validation of CTIMS from a range of different perspectives.

## Methods

### Extraction of Regulatory Requirements

Based on the 2018 FDA imaging guidance, which is the most important regulation covering a wide range of imaging processes required in clinical trials, we extracted key functions of CTIMS to perform imaging processes and enhance efficient workflow in the clinical trials.

**Figure 1:**
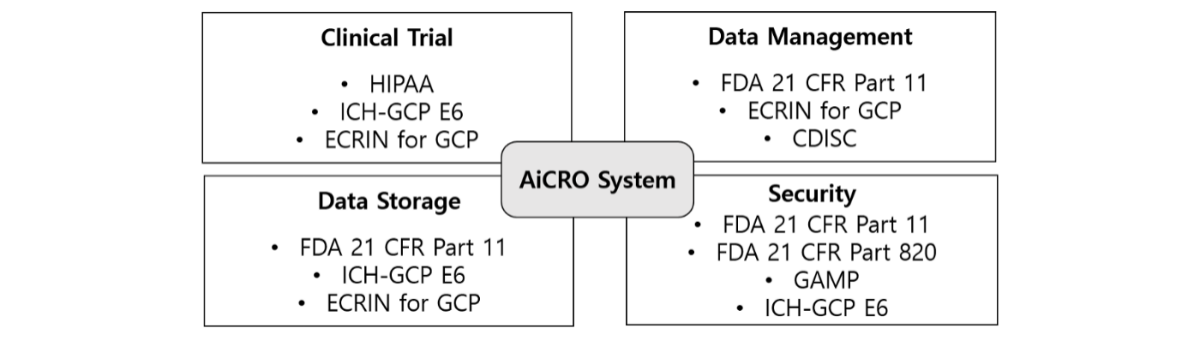
Guidelines and standards related with the AiCRO system. In the four categories of regulatory requirements of CTIMS, the major regulations/guidelines include FDA 21 CFR Part 11, FDA 21 CFR Part 820, ICH-GCP E6, and GAMP 5 guide for compliant GxP computerized systems, ECRIN standard requirements for GCP, CDISC, and HIPAA. ICH-GCP: International Conference on Harmonization Good Clinical Practice; GAMP: Good Automated Manufacturing Practice; CDISC: Clinical Data Interchange Standards Consortium; HIPAA: Health Insurance Portability and Accountability Act; FDA: Food and Drug Administration; ECRIN: European Clinical Research Infrastructures Network; CFR: Code of Federal Regulations.

To systematically extract key GxP requirements, which are not described in the 2018 FDA imaging guidance, we thoroughly reviewed the related regulations/guidelines from regulatory agencies and international societies (Figure 1) as follows:

- Regulatory agency: ICH-GCP E6 (R2) [10], HIPAA, FDA Guidance for Computerized System Used in Clinical Trials [15], or 21 Code of Federal Regulations (CFR) Part 11 [16], etc.
- International society: Good Automated Manufacturing Practice (GAMP) 5 Guide for Compliant GxP Computerized Systems [17], European Clinical Research Infrastructures Network standard [18], good practice for computerized systems in regulated GxP environments, Pharmaceutical Inspection Co-operation Scheme Inspectors Guide [14], etc.

### Development of AiCRO System

Based on extracted regulatory requirements, we designed the AiCRO system architecture and functional modules. Java and HTML5 programming languages were used to develop a platform-independent system, which can be executed on a standalone system or Web-based system regardless of the operating system. The image viewer in AiCRO system was developed using JDK [computer software] (Version 1.8.1. Redwood City, CA: Oracle Redwood Shores).

A multidisciplinary software development team was organized, including radiologists, imaging technicians, clinical trial specialists, regulatory medicine professionals, and IT engineers, who worked toward the same goal. We also recruited potential AiCRO system users, including medical researchers, and pharmaceutical company staff.

### Computerized System Validation

**Figure 2:**
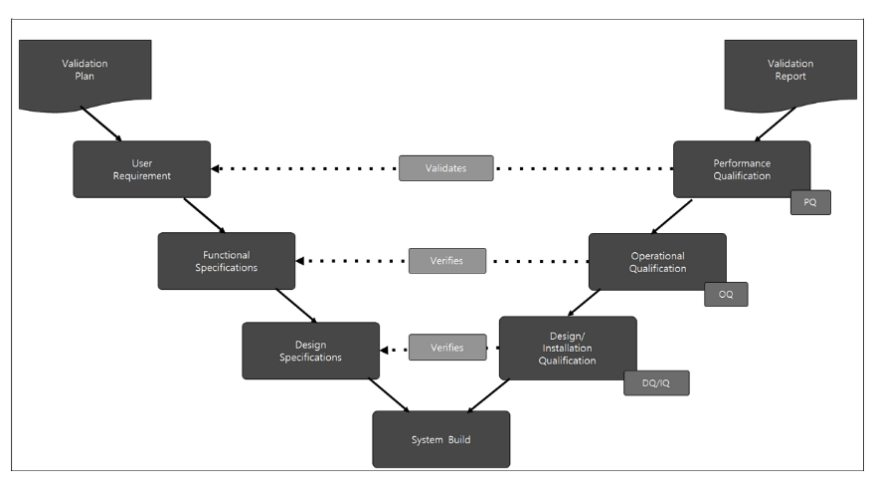
Computerized system validation process. IQ: installation qualification; OQ: operational qualification; PQ: performance qualification. DQ: design qualification.

The AiCRO system was validated both internally and externally. We validated the AiCRO system internally using the V-model in accordance of the US FDA CFR 21 Part 820 and GAMP 5 guidelines, which are currently regarded as the global standards for computerized system validation for clinical trials (Figure 2) [17]. According to the GAMP 5 guidelines, the validation process of the AiCRO system included the installation qualification, operational qualification, performance qualification, and design qualification. Installation qualification refers to the verification of installation and configuration of software and hardware based on preapproved specification.

Operational qualification and performance qualification verify that a system operates and performs all activities under normal and challenging conditions. Design qualification verifies individual system designs for different projects to ensure that they proceed efficiently through the work process. Internal validations are to be performed once a year using at least 10 personal computers in Korea, the United States, China, Australia, and France. For test purposes, we built a sample project that requires typical work processes used in clinical trials for cancer. The AiCRO system was externally validated for global clinical trial use by a global quality assurance and auditing company. Use of a third party enabled different perspectives on our system, including those related to adherence to regulatory requirements.

## Results

### Key Regulatory Requirements

The key regulatory requirements extracted through our systematic review of the FDA guidelines and regulations are summarized in Table 1. The functional requirements for imaging processes in clinical trials are discussed in detail in the 2018 FDA imaging guidance [8]. The ultimate goal of the guidance is to increase assurance of imaging data for analyzing drug efficacy. The guidance comprehensively streamlines imaging processes such as study startup, standardization of image acquisition/interpretation, site monitoring, reader management, document management, image data management, image analysis/review, and data report and export. Of these, several processes require the CTIMS functions, particularly for image data management such as anonymization, transfer, archiving and storage; image analysis/review such as image viewer, electronic case report form (eCRF), and independent review workflow; and the data report and export (Table 1).

The FDA has continuously emphasized and recommended compliance with regulations for computerized system use in the clinical trial, including the FDA 21 CFR part 11 and many other regulations, as illustrated in Figure 1. In addition, the CTIMS must comply with the clinical trial regulation such as privacy protection law. In order to statistically analyze the data with the standardized data structure at the end of the trial, CTIMS should adapt the CDISC format, as described in the Modules section below (Electronic Case Report Form Data Management).

**Table 1:** Regulatory requirements for the Clinical Trial Imaging Management System.

Imaging process			Regulatory requirements		Functional requirement
**Study startup**					
	Imaging protocol set up and imaging charter development	Validation: Computerized systems should be validated for product quality and safety and data integrity. Recorded data are recommended to be standardized to submit to regulatory agencies for review.		Provision of templates: When researchers establish the imaging protocol and develop the imaging charter for starting a new clinical trial, the CTIMS^a^ provides several templates of typical imaging process and workflow, so that the researchers can tailor the template according to the new project.	
	CTIMS set up	Validation: Computerized systems should be validated for product quality and safety and data integrity. Recorded data are recommended to be standardized to submit to regulatory agencies for review.		User requirement specification: In order to easily set up the CTIMS for the new clinical trial, CTIMS offers electronic forms of user requirement specification, allowing researchers to easily fill the form.	
**Image data management**					
	Deidentification	Deidentification: Personal health information for both patients and participants should be protected.		DICOM^b^ file deidentification: CTIMS provides a function to anonymize the imaging data. If there are only DICOM files, CTIMS removes several items of patient’s information from the DICOM metadata. Graphical image deidentification: If there are images with graphically inserted patient information, CTIMS provides a function to graphically remove that information.	
	Transfer/archiving/storage archiving/storage	Back up: Data should be regularly backed up to protect from any system attacks or unpredicted circumstance.		Secure file transfer function: CTIMS must provide a function to transfer image files through network from site to central server in secure ways. Archive of electronic data: CTIMS provides secure storage spaces for archiving electronic data and allows only authorized personnel to access the data.	
	Image QC^c^	Standards for image acquisition: During the clinical trial, image acquisition should be standardized and involve imaging modalities, equipment operation in each site, and image quality.		Image QC: Automatic and manual image QC functions for image analysts are to check the image quality and decide appropriateness of an image for image review or analysis.	
**Image analysis/review**					
	Image viewer	Copies of records and record retention: Data should be retained in either electronic or nonelectronic format. Digital signature: It refers to a legal mark and is equivalent to individual handwritten signature for adapting the present intention.		DICOM image viewer: CTIMS provides functions to view medical images and is in a DICOM or another file format. It also has a tool to measure lesion size, area, or volume.	
	Electronic CRF^d^	Copies of records and record retention: Data should be retained in either electronic or nonelectronic format. Digital signature: It refers to a legal mark and is equivalent to individual handwritten signature for adapting the present intention.		Image CRF: When central independent reviewers analyze the image for clinical trials, the analysis results can be recorded electronically in the CTIMS.	
	Centralized imaging review workflow	Security system (authorized access): System should ensure to maintain security system to restrict unauthorized access for data protection.		Customizable workflow: CTIMS should provide several functions that are required for central independent imaging review process including blinding, automatic calculation, and adjudication.	
**Report and export**					
	Tracking report	Audit trail: All entered data should be tracked including time stamped.		Master tracking report: For audit trail, CTIMS should provide a report containing all log and activity of a project in a straightforward manner. If there are any modifications in the data, CTIMS should track the modification of data and record of the reason.	
	Data export	CDISC^e^: All data should be in a standardized data format.		Review data export: The image review/analysis results are exported and transferred to the DM^f^/statistics team in a global standardized data format such as CDISC. Image QC results export: CTIMS provides a function to report image QC results. If there are queries, this report should include all queries and consequences of queries such as query resolution or protocol violation.	

^a^CTIMS: Clinical Trial Imaging Management System.

^b^DICOM: digital imaging and communications in medicine.

^c^QC: quality control.

^d^CRF: case report form.

^e^CDISC: Clinical Data Interchange Standards Consortium.

^f^DM: Data Management.

### Overall AiCRO System Architecture

**Figure 3:**
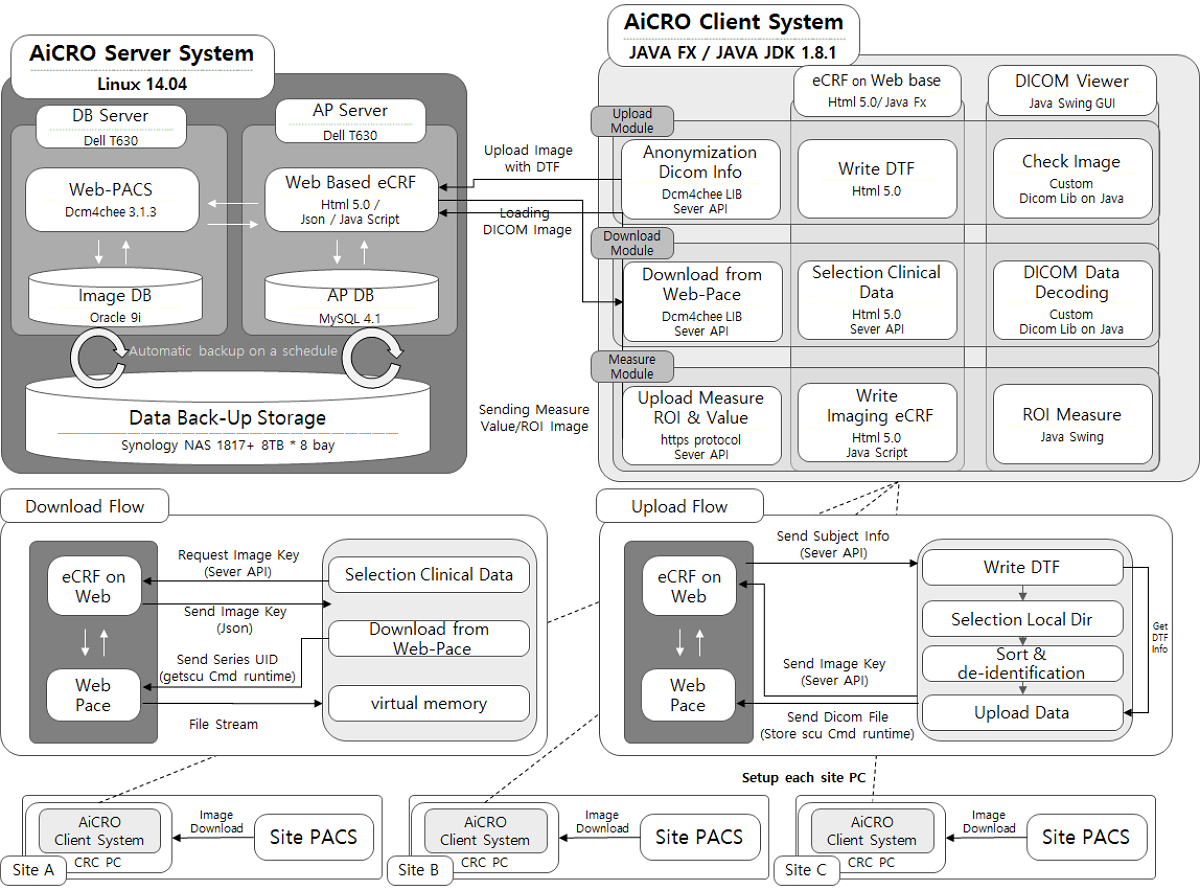
System architecture of the AiCRO system. AP: application programming; API: application programming interface; eCRF: electronic case report form; DICOM: digital imaging and communications in medicine; PC: personal computer; CRC: clinical research coordinator; NAS: Network-Attached Storage; DB: database; PACS: picture archiving and communication system; UID: unique identifier; DTF: data transfer form.

The predominant factor in the system design was compliance with a diverse set of regulations. We developed the AiCRO system for imaging processes in clinical trials in strict accordance with the relevant guidelines, with a focus on FDA 21 CFR Part 11 and the FDA imaging guidance. AiCRO system is developed based on Server and Client system architecture, as illustrated in Figure 3. The server system was organized with the database server, a Web-Picture Archiving and Communication System (PACS) based on dcm4chee, the AP server for Web application of eCRF, and Network-Attached Storage (NAS) for data backup. The database server was defined to handle large DICOM image files. It archives only DICOM images and provides functional modules to upload and download images to rest application programming interface (API). On the other hand, the AP server archives thumbnails or image measurement values provided by reviewers and eCRF text data along with API for these processes.

**Figure 4:**
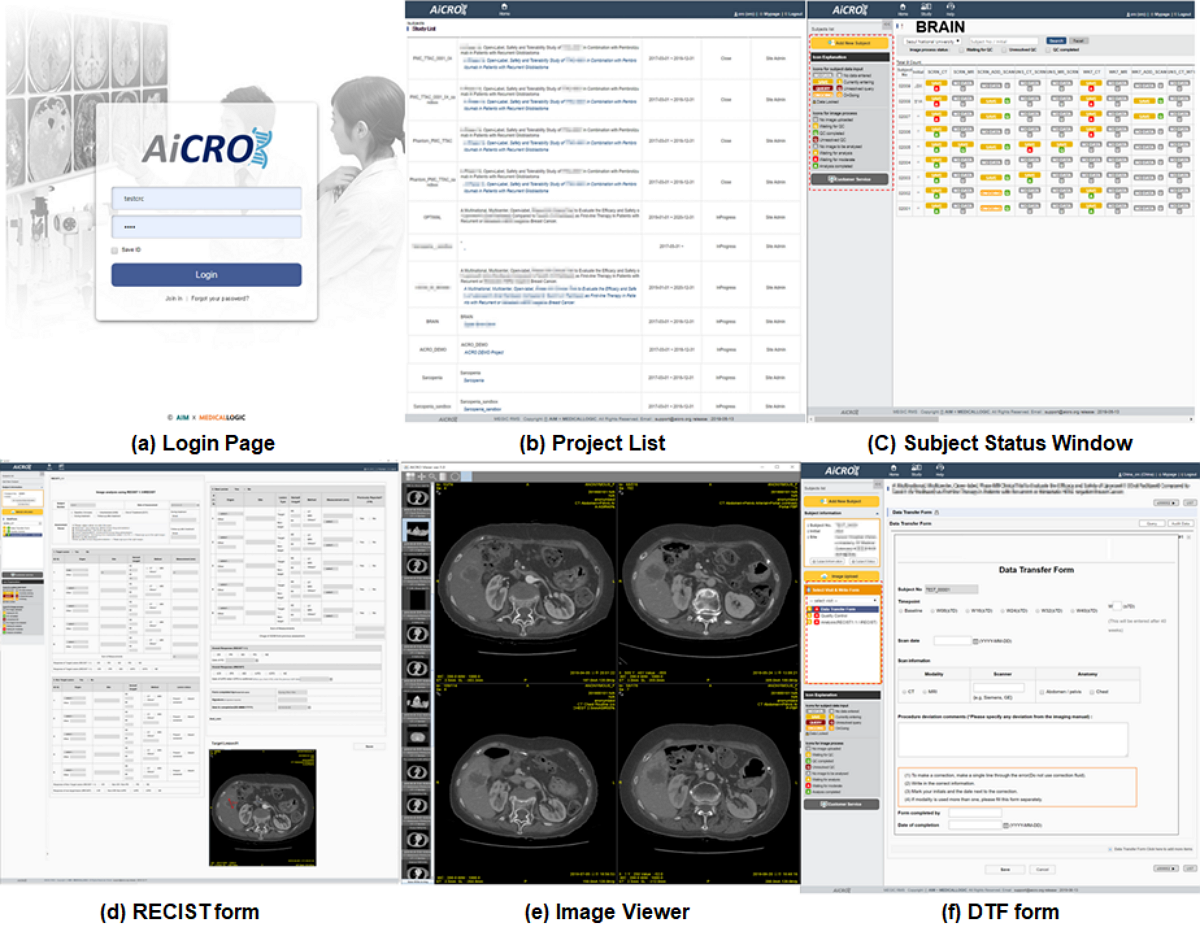
AiCRO system snapshot. RECIST: response evaluation criteria in solid tumors; DTF: data transfer form.

The client system was designed with various modules to manage clinical information data. The system transfers the eCRF clinical data to an AP server via API and transfers images to the server through database API after the image data are deidentified. Reviewers can also check the uploaded DICOM image with the image viewer and analyze images to, for example, measure the tumor size or volume. These measurement values are delivered to the AP server by the client system. If the client system is installed on the user’s laptop, clinical data can be easily managed and images can be viewed and analyzed from any location. The screen snapshots of modules are presented in Figure 4.

### Modules

#### Image Upload With Deidentification

Every medical image acquired from the participating sites should be uploaded to the AiCRO system in DICOM format by each site’s staff. Uploaded image data are anonymized, transferred, and archived in the central server of CTIMS [20]. Deidentification of medical images is one of the most important components for GxP compliance with regard to GCP and the HIPAA privacy guideline [8,10].

Personal identification information is recorded in the DICOM file metadata or presented in the medical images as graphical information. The metadata contains both patient identification information such as patient name; date of birth; hospital name; and hospital medical record number; and imaging modality/protocol information such as manufacturer and software of the modality, sequence, field of view, or slice thickness. According to the DICOM standard part 15 [21], the AiCRO system extracts DICOM elements containing personal identification information, automatically removes the extracted information, and asks uploaders to enter alternative information such as the subjects’ screening number or clinical participant code (Tables 2 and 3). However, some imaging machines also use private DICOM elements to store complementary information such as patient or physician initials, telephone number, or national identification number. We created a function that would detect any potential patient identification information from DICOM metadata by using keywords and number formats.

Besides metadata in the DICOM file, patients’ identification information is presented on medical images as graphical texts or image pixel data, especially in the ultrasonography, 3D rendering computed tomography images, radiation dose reports, screen shot images, and endoscopy images. We implemented two functional modules to remove this type of information by incorporating the fully automatic text-removal function and semiautomatic imaging-processing blackout function.

Specifically, graphical patient identification information was detected through the incorporation of optical character recognition, which is based on the convolutional recurrent neural network algorithm to detect text burned in the images [22]. Subsequently, we used a rule-base text recognition module to detect patients’ name, date of birth, medical record number, and institution name. However, it might not be accurate and therefore requires the uploaders to check the deidentified images. If further action is required, a manual imaging-processing function can be applied to hide such information.

**Table 2:** The deidentification process (Part 1). The AiCRO system identifies items and performs deidentification actions by predefined action code.

Action code	Intended action
D	Metadata of name and ID^a^ are replaced with word “DE-IDENTI.”
R	Metadata of scan/birth date are replaced with format “0000-0000.”
E	The original metadata are removed.
C	The original UID^b^ metadata are replaced with new UID.
N	A specific word is replaced with a new dedicated word.

^a^ID: identification.

^b^UID: user identification.

**Table 3:** The deidentification process (Part 2).

Attribute name	Tag	Action code	Example
Patient name	0010, 0010	N(Initial)	Jhon Doe → patient01
Media storage SOP^a^ instance UID^b^	0002, 0003	C	1.3.12.2.1107... → 1.2.410.200...
SOP instance UID	0008, 0018	C	1.3.12.2.1107... → 1.2.410.200...
Accession number	0008, 0050	E	00009 → Remove
Institution name	0008, 0080	D	AMC^c^ → “DE-IDENTI”
Institution address	0008, 0081	E	88, Olympic-ro 43-gil...→ Remove
Referring physician name,	0008, 0090	E	Jane Doe → Remove
Performing physician name	0008, 1050	E	Jane Roe → Remove
Patient ID^d^	0010, 0020	N(Subject NO)	11112222 → Subject01
Patient’s birth date	0010, 0030	R	19890215 → 19890101
Other patient names	0010, 1001	E	Jhon Roe → Remove
Other patient IDs	0010, 1002	E	22223333 → Remove
Patient address	0010, 1040	E	17, Misagangbyeon… → Remove
Study ID	0020, 0010	N(Project ID)	33334444 → Project01
Study instance UID	0020, 000D	C	1.3.12.2.1107... → 1.2.410.200...
Series instance UID	0020, 000E	C	1.3.12.2.1107... → 1.2.410.200...

^a^SOP: standard operating procedure.

^b^UID: user identification.

^c^AMC: Asan Medical Center.

^d^ID: identification.

**Figure 5:**
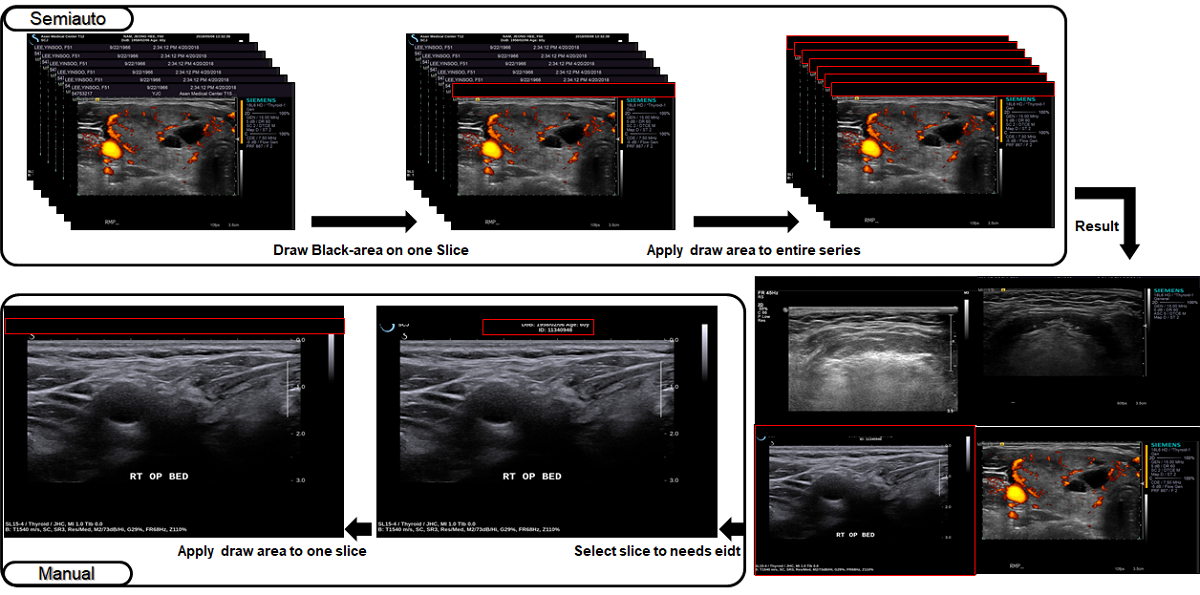
Semiautomatic/manual blackout deidentification function. The users select blackout regions, which will be filled with black pixels. If similar image formats are used in a specific project, users can predefine the blackout regions and remove the regions semiautomatically. If further action is required, the manual blackout function can be applied.

The semiautomatic and manual blackout deidentification functions enable uploaders to select regions of interest that need to be removed (so-called blackout regions) either in a single image or multiple series of images (Figure 5). If the blackout function is successfully applied, the processed images are stored in new anonymized DICOM files and then uploaded into the AiCRO system.

#### Centralized Imaging Data Storage and Security

Data storage and security are other significant points emphasized by FDA guideline 21 CFR part 11. Hence, all DICOM files are centralized and stored on the AiCRO server system (Figure 3). Web-PACS of AiCRO system was developed using the dcm4che library [23], and Oracle 9i was developed for storing DICOM files. The eCRF data are stored in the AP server, which was built with HTML 5.0 and the MySQL 4.1 database. Two separate databases were used because, although Oracle enables large-scale databases to store large DICOM files, MySQL is better for managing smaller amounts of data such as eCRF text data.

Most hospitals and institutions use DICOM metadata tagging in accordance with DICOM standards, but specific information therein may vary across sites depending upon the institutional policy. If the metadata does not follow standard DICOM protocols, image upload and view may be disabled by the Web-PACS. We resolved this issue by developing a custom DICOM library for the standardization of DICOM metadata. The custom DICOM library enables creation of new metadata by modifying the pre-existing DICOM metadata. These changes are stored as per the custom DICOM library, which must be regularly updated to apply to different or emergent circumstances.

Regarding a system security to prevent different types of system attack, we established an IT system security plan. For example, separation of the AP server and database server can localize any damage in the server. To protect the local computer, we adopted a zero footprint design and symmetric encryption algorithms based on advanced encryption standards. Images can be only loaded into our custom-built DICOM viewer due to the encryption key, and there are no plugins or data in the local personal computer.

To protect against any unforeseen disaster (fire, inundation, or hack), backup data are stored regularly with a dedicated program using NAS hardware physically located at different sites. When any issue arises, the server administrator receives an automatic email alert with the relevant information for troubleshooting. Backup data include the database, audit trails, DICOM, and configuration files. These overall securities allow us to guarantee data integrity.

#### Image Quality Control

All uploaded medical images should pass through quality control (QC) before image analysis in accordance with the FDA imaging guidance [8]. The image QC module in the AiCRO system can be customized according to the predefined imaging acquisition protocols and QC criteria for each clinical trial [24].

Our image QC module is composed of an automatic quantitative QC module and a manual qualitative QC module. The automatic quantitative QC module extracts several DICOM elements containing imaging modality/protocol information such as machines, sequences, matrix size, spatial resolution, field of view, slice thickness, and interslice gap. The module automatically decides whether the extracted values meet the predefined image quality requirements. For instance, if a trial requires axial and coronal computed tomography images with a matrix size of 512 × 512 and slice thickness of ≤5 mm with 0 interslice gap, the automatic quantitative QC module checks whether the uploaded images meet those specific requirements.

**Figure 6:**
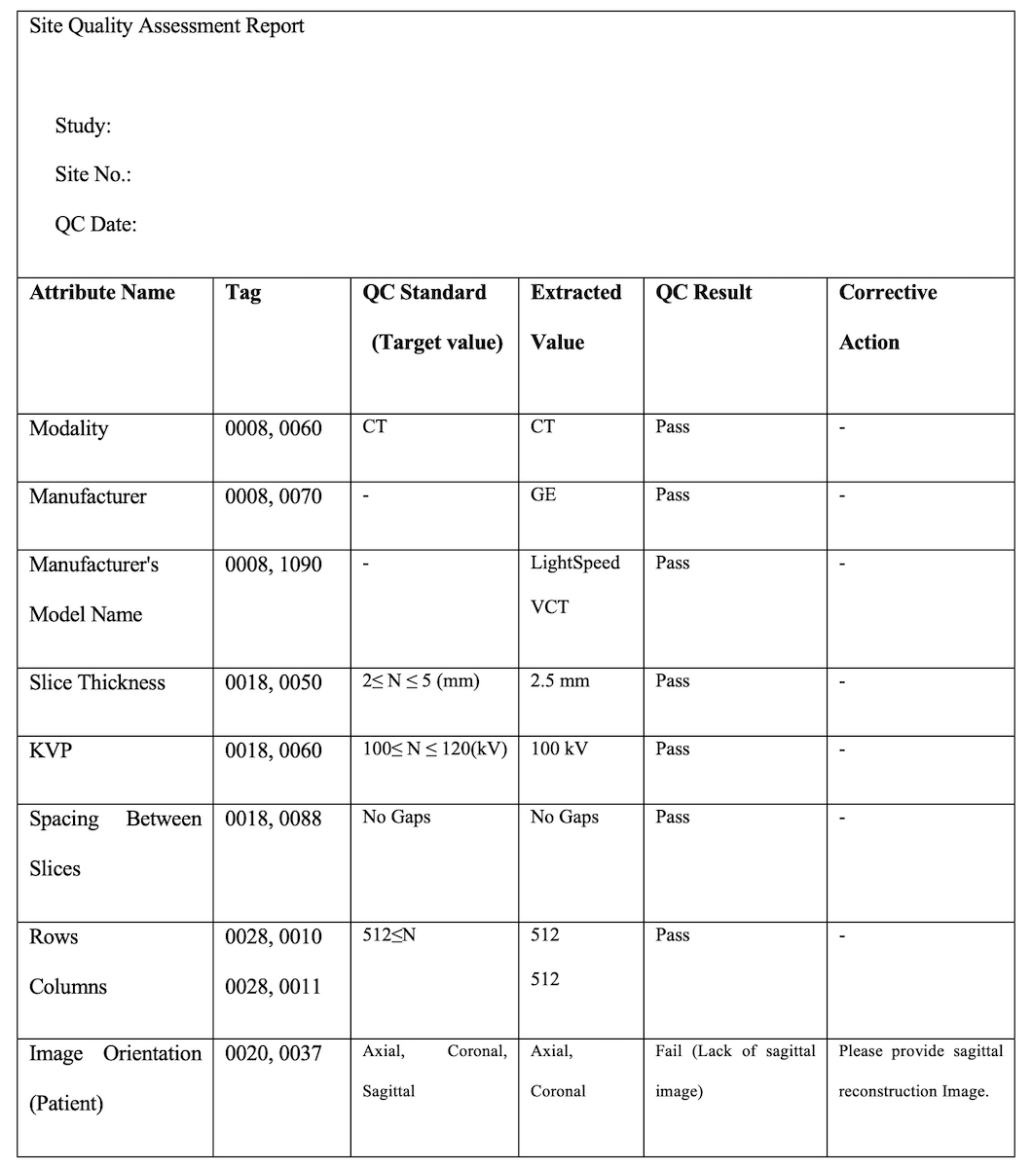
Quality assessment report form. QC: quality control; CT: computerized tomography; KVP: Kilovoltage.

Image QC staff is involved in the manual qualitative QC module. The AiCRO system supports image QC staff to perform qualitative image quality checks for presence of image artifacts, appropriateness of scan coverage, image reconstruction, and contrast enhancement. The image QC staff can generate the quality assessment report after the QC check (Figure 6). The quality assessment report includes the quantitative QC results, qualitative QC results, overall quality to decide pass or fail, detailed information on any protocol deviations, queries for corrective action (if necessary), and query resolution.

#### Image Viewer

The AiCRO image viewer was developed to provide a viewer platform, interfaces, and image processing tools [24]. Our viewer supports DICOM files obtained from common image modalities such as ultrasound, endoscopy, computed tomography, magnetic resonance, or positron emission tomography. The configuration function is provided to help the user quickly configure viewer setting customized for the trial with encrypted XML file. The time for image loading is minimized by using multithread processing, as this enables viewing images while others are loading, eventually reducing working time. We also incorporated a multiplatform function into the AiCRO image viewer, enabling both standalone and Web-based image viewing, using Java to develop platform-independent software that can be executed on Windows or Linux operating systems using Java runtime environment or on a Web-based viewer using Java Web Start.

As there are already established image processing tools in most image viewers (Table 4) [25], we made all such tools available in the AiCRO image viewer; this viewer provides central image reviewers a range of tools for manipulating images in the AiCRO system. Measurement tools for region of interest (ROI) are also implemented, assisting reviewers in measuring key areas on uploaded images. Once measurements are made, the ROI image and measure value can be automatically saved and recorded into the imaging CRF in the AiCRO system. Since the ROI image and measure value are saved in the system, subsequent reviewers can retrieve this information for reference purposes and analysis of additional images. Other necessary reviewing functions include image contrast control and zoom. We also developed a multipotent imaging postprocessing software, called Asan-J software, that enables important postprocessing functions such as image registration, subtraction, semiautomatic segmentation, image classification, 3D rendering, and functional image analysis.

**Table 4:** Image viewer function list.

Function	Detail
Scrolling and positioning	Move between images by scrolling with the mouse wheel Automatically repositions all images in the same orientation when viewing several at once
Metadata	Header viewing in table format, including private headers
Information overlay	Important information visualized in view panel as an overlay
Windowing	Windowing for control of brightness and contrast of the displayed image; presets supported with hotkeys
Measurements	Allows drawing, distance, area, and angle
Histogram	Advanced mode using AsanJ
Pseudocolor	Advanced mode using AsanJ
3D image viewer	Advanced mode using AsanJ

#### Electronic Case Report Form Data Management

All textual data including subject information and image analysis and review results in the AiCRO system adhere to CDISC standards for data formats for multicenter and multinational trials, a prerequisite for FDA submission. The FDA insists on the use of study data standards for data consistency, particularly in computer systems [26-28].

A diverse and customized eCRF, also known as an imaging CRF including Response Evaluation Criteria in Solid Tumors (RECIST) 1.1, Immune RECIST, or Lugano classification, was developed to retain records of all data related to study images. Customized eCRFs were created for different study designs by anatomical location of tumor, tumor measurement, or type of drug or analysis, leading to inherent variations in data formats. We streamlined data formats according to the CDISC terminology for each therapeutic area, such as cardiology, neurology, or oncology, so that all data are collected using standardized CDISC terminology from the start to enable data management across the sites. These data collection process adhere to the Clinical Data Acquisition Standards Harmonization of CDISC standards [24].

Collected data in the system need to be organized to standard structures for submission to regulatory agencies, as discussed in the CDISC standards, in the study data tabulation model [27]. Implementing an appropriate data standard for data collection and export allows for a variety of perspectives on clinical and nonclinical trial data. At the end of the trial, standardized data save time in terms of converting the data for statistical analysis and compiling a final report to submit to the FDA. Creation of standardized analysis datasets for metadata is included in the Analysis Data Model of CDISC standards [28].

**Figure 7:**
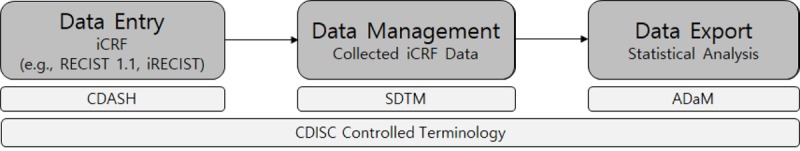
AiCRO system eCRF data management process following CDISC Standard. eCRF: electronic case report form; CDISC: Clinical Data Interchange Standards Consortium; RECIST: response evaluation criteria in solid tumors; iRECIST: immune response evaluation criteria in solid tumors; iCRF : imaging case report form; SDTM: study data tabulation model; ADaM: Analysis Data Model.

AiCRO complies suitable CDISC terminology for all these processes (Figure 7), which are already set up for eCRF from the start of the trial and manage data from all sites for standardization. CDISC-controlled terminology transforms vocabulary used in clinical trials such as demographic information, adverse events, and medical examination from study protocols to CDISC codes or values. Consistency is critical when developing data integration systems, and all data in the AiCRO system are optimized for submission to the FDA in the United States and Pharmaceuticals and Medical Devices Agency in Japan without any additional processing.

#### Electronic Signature

As the AiCRO system maintains electronic data, data validation is an important consideration for clinical trials. Data validation in an electronic system is mandated by regulations specific to electronic signatures. We thereby incorporated an electronic signature function into the system, as per FDA 21 CFR part 11, wherein electronic signatures must be “the equivalent of handwritten signatures, initials, and other general signings required by predicate rules” [29].

#### Audit Trail

FDA 21 CFR part 11 [29] stipulates that all data are to be recorded with computer-generated, time-stamped audit trails representing date, time, editor, and any subsequent modifications. Thus, we provide for this in the AiCRO system. The guideline also emphasizes that, as in handwritten medical records, any changes to data must not obscure previous data, and the AiCRO audit trails are thus situated: Audit trails are generated whenever data are created, modified, or deleted during the clinical trial period and after trial closeout. These audit trail data can be extracted upon requests for inspection of records by other stakeholders such as pharmaceutical companies, CROs, or ministries of health.

#### Query and Alarm

The query and alarm functions are designed to adhere to GAMP guidelines for corrective and preventive action (CAPA). CAPA outlines a process for the investigation, discussion, and correction of any problems as well as recognition and prevention of potential problems [17]. When designing a system suitable for use in clinical trials, the AiCRO system CAPA utilized the term “Query,” as it is commonly used in such trials. Generated queries from the AiCRO system fall into two categories: system problems such as image upload issues and clinical trial problems such as missing data.

Appropriate data entry is key in clinical trials and usually managed via “edit check,” a process for the detection and correction of data when they are first entered [24]. The “Query” function is engaged during the QC process in reference to image quality or mismatch of imaging modality or protocol and during monitoring by the clinical research associate in cases of site data omission from the CRC or central reviewers. The function sends the generated query to the person responsible for the trial to allow him/her to resolve issues and is structured similarly to commonly used email systems.

#### Role and Responsibility of Account

Accounts for accessing the AiCRO system are organized into different types depending on the individual’s role, such as site CRC, image QC, central reviewer, and many others. According to GAMP guidelines, definitions of the roles and responsibilities attached to each account are recommended for validation purposes, including the testing of computerized systems [17]. The AiCRO system administrator gives suitable permissions to different personnel and is responsible for overall account management over the study period. This function serves two purposes. From a clinical trial perspective, it blocks access to information that could affect imaging analyses among central reviewers, ensuring impartial review outcomes. From a computerized system perspective, this function allows the administrator to test the system for appropriate plans or test strategies. Parsing out permissions depending on the account enables improved system development and efficient control over clinical trial data.

### Computerized System Validation

For internal system validation, we organized the overall validation plan according to the V-model (Figure 2), which includes the purpose, process validation, test setting and methods, and schedule. All validation processes and results were documented.

For installation qualification, the AiCRO’s client system was successfully installed in all test personal computers, regardless of the operating system such as Windows and Mac as well as Web-browsers including Chrome, Internet Explorer versions 10 and 11, and Firefox. The AiCRO’s server system was installed appropriately on our institutional server and Amazon Web Services Clouds in Seoul and China (Beijing).

For operational qualification, the system developer created test scripts to prove the proper functioning of all functional specifications and system operation. Subsequently, an independent tester executed test scripts in a predefined testing environment and recorded the test log and all results including test date, observations made, completeness of each function, problems/deviations encountered, and other information relevant to the operational qualification. These assessments found that all AiCRO functions could be operated without difficulty.

For performance qualification, we prepared a sample project with real data and different user accounts. An independent tester then evaluated module function using the sample project with different amounts of imaging data, work load sizes, and computing and network environments. All functions of the AiCRO worked appropriately under normal operating conditions of minimum required personal computer specifications, and the upload and download of several computed tomography/magnetic resonance imaging scans was performed at usual internet speeds of over 10 megabytes per second. However, during testing under challenging conditions, limiting factors that might cause an interruption or significant delay in activity were identified, which were machines with less than 4 GB RAM, uploading and downloading large image data for at least 3000 DICOM files at one time, internet speeds below 10 MB per second, and viewing large image data for at least 3000 DICOM files at once.

For design qualification, AiCRO validation is essential because each clinical trial project will have a different study design. We checked whether AiCRO was able to create a customizable data entry form and working algorithm per user specifications. Using a test project that simulated a sophisticated clinical trial for stroke, our imaging clinical research associate wrote the user specification, and our system developer created the customizable project in the AiCRO system. Thereafter, a test team, including a project manager, image analyst, and radiologist, performed user acceptance tests. The customized test project was successfully created, and its functional modules garnered acceptance from different kinds of users.

Finally, the AiCRO system was validated by Zigzag (Berkshire, United Kingdom), an external global quality assurance company. A professional quality manager from Singapore visited the core lab in Seoul for 3 days and reviewed all validation documents. This manager also required a demonstration of the process, including creation of a new customized project and associated functions. The quality manager verified that installation qualification, operational qualification, performance qualification, and design qualification were performed appropriately in accordance with the relevant regulations and guidelines.

## Discussion

### Principal Findings

During the 2000s, the FDA [4] was concerned about the objectivity and validity of imaging endpoints, since there were several critical issues such as reliability of imaging biomarker and results, and appropriateness of imaging data management with regard to GCP, FDA guidelines, and HIPAA. The FDA addressed this issue by developing the Clinical Trial Imaging Endpoint Process Standards Guidance for Industry. The first draft guidance was issued in 2011, with a revision in 2015 and finalization in 2018 [8]. The guidelines emphasize adherence to regulations for imaging data collection, data transfer, and imaging analysis. At present, all stakeholders including pharmaceutical companies, imaging scientists and clinical trial professionals, and hospitals are expected to follow the 2018 FDA guidance to conduct clinical trials successfully.

CTIMS thus needed to comply with FDA guidelines, which comprehensively cover the functional and regulatory aspects of imaging, thereby underscoring the importance of developing a CTIMS that was GxP-compliant. The appropriate use of medical imaging in clinical trials necessitates specific considerations including standardization of imaging acquisition, archiving, and QC; centralized independent blinded image review; and systems that can handle complex workflows while maintaining regulatory compliance.

Asan Image Metrics (Seoul, Korea), an academic imaging core lab, developed a CTIMS—the AiCRO system—which adheres to all regulatory requirements for medical imaging in clinical trials and enables stakeholders to access easily medical images obtained from different sites. It contains modules including deidentification, data transfer, data archive, eCRF, image viewing/analysis, data management according to CDISC, audit trail, query/alarm, complex workflow algorithm, and security. The platform thus ensures quality of results and minimizes data risks during and after clinical trial periods. In the last 2 years, we have implemented the AiCRO system on more than 20 domestic and international pharmaceutical multicenter clinical trials, and several audits and inspections have been conducted as well.

### Strengths and Limitations

The main advantage of AiCRO is that it is an all-in-one system that meets virtually all regulatory and functional requirements for clinical trial imaging while maintaining considerable user flexibility. For example, if a user needs a new function for the specific trial, our development team can easily build or customize the necessary function according to user specifications, after which our quality assurance and data management teams offer continuous user and regulatory support for the new function. For example, in the cases of trials on rare diseases requiring sophisticated magnetic resonance imaging analyses, a dedicated imaging postprocessing module and disease-specific terminology can be added in the CDISC data format.

Although our system has several advantages, one of the limitations is that each site or hospital has its own security system containing firewalls to protect their patients’ information. Nowadays, many institutions strictly regulate the external transfer of medical data, and hospital network system firewalls are becoming stronger. Ideally, a CTIMS should strike a balance between security and function within the boundaries of regulations and institutional policies. Another limitation is that AiCRO is an independent system separated from other EDC system. Thus, in clinical trials, we need to link our system to other EDC systems. Otherwise, users have to use two different IT systems in a clinical trial.

### Conclusions

We introduced a CTIMS, called AiCRO system, to conduct clinical trials more efficiently in accordance with regulatory requirements. In this paper, we discussed the development of the AiCRO system to meet a diverse array of regulatory requirements and the design of multiple modules for users to customize the system to the needs of their clinical trials. We also discussed the internal and external validation of AiCRO according to the GxP guidelines and FDA 21 CFR. The AiCRO system is an all-in-one platform enabling high-quality clinical trial imaging data, but further study is required to describe the results of implementation obtained for different types of trials and any necessary systemic improvements.

## Abbreviations

API: application programming interface
CAPA: corrective and preventive action
CDISC: Clinical Data Interchange Standards Consortium
CRO: clinical research organization
CTIMS: Clinical Trial Imaging Management System
DICOM: Digital Imaging and Communications in Medicine
eCRF: electronic case report form
EDC: electronic data capture
FDA: Food and Drug Administration
GAMP: Good Automated Manufacturing Practice
GCP: Good Clinical Practice
HIPAA: Health Insurance Portability and Accountability Act
ICH: International Conference on Harmonization
IT: information technology
NAS: Network-Attached Storage
PACS: picture archiving and communication system
QC: quality control
RECIST: Response Evaluation Criteria in Solid Tumors
ROI: region of interest
